# Intrarenal Reflux in the Light of Contrast-Enhanced Voiding Urosonography

**DOI:** 10.3389/fped.2021.642077

**Published:** 2021-03-02

**Authors:** Ana Simicic Majce, Adela Arapovic, Mirna Saraga-Babic, Katarina Vukojevic, Benjamin Benzon, Ante Punda, Marijan Saraga

**Affiliations:** ^1^University Hospital Split, Split, Croatia; ^2^School of Medicine, University of Split, Split, Croatia

**Keywords:** vesico ureteral reflux, intrarenal reflux, contrast-enhanced voiding urosonography, kidney, children

## Abstract

**Purpose:** The aim of this study was to analyze the incidence of intrarenal reflux (IRR) among vesicoureteral refluxes (VURs), diagnosed by contrast-enhanced voiding urosonography (ceVUS), to define VURs which are positive to IRR and their locations in the kidney.

**Materials and Methods:** Seventy patients with VURs, including 103 uretero-renal units (URUs) with VURs of grades II–V (37 URUs were excluded because of renal anomalies or absence of VUR) were examined with ceVUS due to recurrent febrile UTI or first febrile UTI accompanied by abnormalities on renal ultrasonography. Patients were examined on GE Logiq S8 ultrasound machine, using second generation of ultrasound contrast agent.

**Results:** Out of 103 VURs, 51 (49.51%) had IRR regardless the grade of VUR, showing increase in IRR incidence with VUR severity (*p* < 0.0001). The median age at the time of IRR diagnosis was 5 months (IQR, 3–14.3), whereas in patients without IRR, it was 15.5 months (IQR, 5–41.5), (*p* = 0.0069). IRR was most common in superior pole (80%), followed by inferior pole (62.7%), and middle segments (37%), and to all segments (27%) (*p* < 0.0001).

**Conclusion:** In the present study, patients with IRR-associated VUR showed earlier clinical presentation. The distribution of IRRs corresponded to the natural distribution of composed papillae types II and III, while the incidence of IRR increased with severity of VUR. Further clinical studies may point to the importance of considering IRR in the future classification of VUR.

## Introduction

Vesicoureteral reflux (VUR) is defined as a backflow of urine from the urinary bladder to the proximal part of the urinary system, including the kidney parenchyma. It is usually diagnosed by voiding cystourethrography (VCUG), direct radionuclide cystography (DRNC), and contrast-enhanced voiding urosonography (ceVUS). It may be associated with an increased incidence of urinary tract infection (UTIs), functional abnormalities of the lower urinary tract (LUT) and bowel, scarring of the kidney parenchyma, and other congenital anomalies of the kidney and urinary tract (CAKUT). Despite a large number of studies, which support the theory that VUR is a very important risk factor for kidney scarring, and the decrease of kidney function, some studies reported opposite results (1, 2). Moreover, those studies recommended that it is not necessary to diagnose VUR, in general. That ongoing debate is still very active and needs new studies to clarify that issue. VUR may reach the kidney parenchyma as intrarenal reflux (IRR), which is defined as backflow of urine into the tubulointrestitial tissue through papillary ducts during VUR. It was first diagnosed in 1965 by voiding VCUG, using barium sulfate as a contrast medium (3). The occurrence of IRR was closely connected to compound papillae, types II and III, while simple papillae and compound papille type I were not associated with the occurrence of IRR (4).

Patients with IRR usually had UTIs and renal scars more frequently than those without IRR (5). Some authors found strong correlation between scars and IRR in a small number of studies and reported scar distribution more frequently in polar areas of the kidney. They concluded that compound papillae types II and III could be the sites of IRRs (6).

The incidence of IRR is ~1–11% in patients with VUR if VCUG is used as the diagnostic method (5, 7–9). Although the existence of IRR itself does not mean the simultaneous existence of parenchymal scar, IRR diagnosed by VCUG showed a high correlation (76%) with defects of radiotracer uptake on dimercaptosuccinic acid (DMSA) scan, suggesting a connection between IRR and scars (8, 9). Moreover, IRR tends to form parenchymal scars in 26–65% of cases over time (5, 10, 11). Boubnova et al. found that the possibility for the breakthrough of urinary tract infections during long-term antibiotic prophylaxis is the same in the group of patients with high grade of VUR and IRR as in those with high-grade VUR but without IRR, whereas some other authors confirmed that patients with IRR had significantly more breakthrough infections than those without IRR (12, 13). It seems that IRR is equally distributed in both genders and that it is present only in patients with higher grades of VURs (III, IV, and V grade), while it has not been observed in patients with lower grades of VURs (grades I and II) on VCUG (9). IRR seems to appear in rather young patients (<4 years of age), while the oldest described patient was 9.5 years old (5, 9). The first case of IRR diagnosed by contrast-enhanced voiding urosonography (ceVUS) was described by Darge et al. in 2003. as entrance of US contrast into the kidney parenchyma, turning the previously hypoechogenic parenchyma in markedly echogenic, indicating IRR, using the first generation of ultrasound contrast agent (UCA) (14).

The second case study was reported on four patients with VUR and IRR by ceVUS, describing IRR by second generation of UCA (15). The new ceVUS procedure with second generation of UCA brought the new possibilities in the diagnostics of VUR and IRR, especially in the grading system in five grades. Some studies reported higher sensitivity of ceVUS compared with VCUG and comparable results with direct radionuclide cystography, regarding the VUR detection (16–23). The aim of this study was to analyze the incidence of IRR among VURs diagnosed by ceVUS, to define the grades of VURs which are positive to IRR, as well as to define their exact IRR locations in the kidney.

## Patients and Methods

### Patients and Data Collection

In this retrospective study, patients with recurrent febrile UTIs or with first febrile UTIs, accompanied with abnormal findings on renal ultrasonography underwent ceVUS, regadless the sex, according to the current recommendations (16, 19–21). The study lasted 3 years (from March 2017 until the end of February 2020). ceVUS is a standard and routine procedure at our Hospital, available to all children with indications for cystography. Since the procedure is minimally invasive, patients usually do not need sedation, which can be rarely applied in some selected patients.

**Figure 1:**
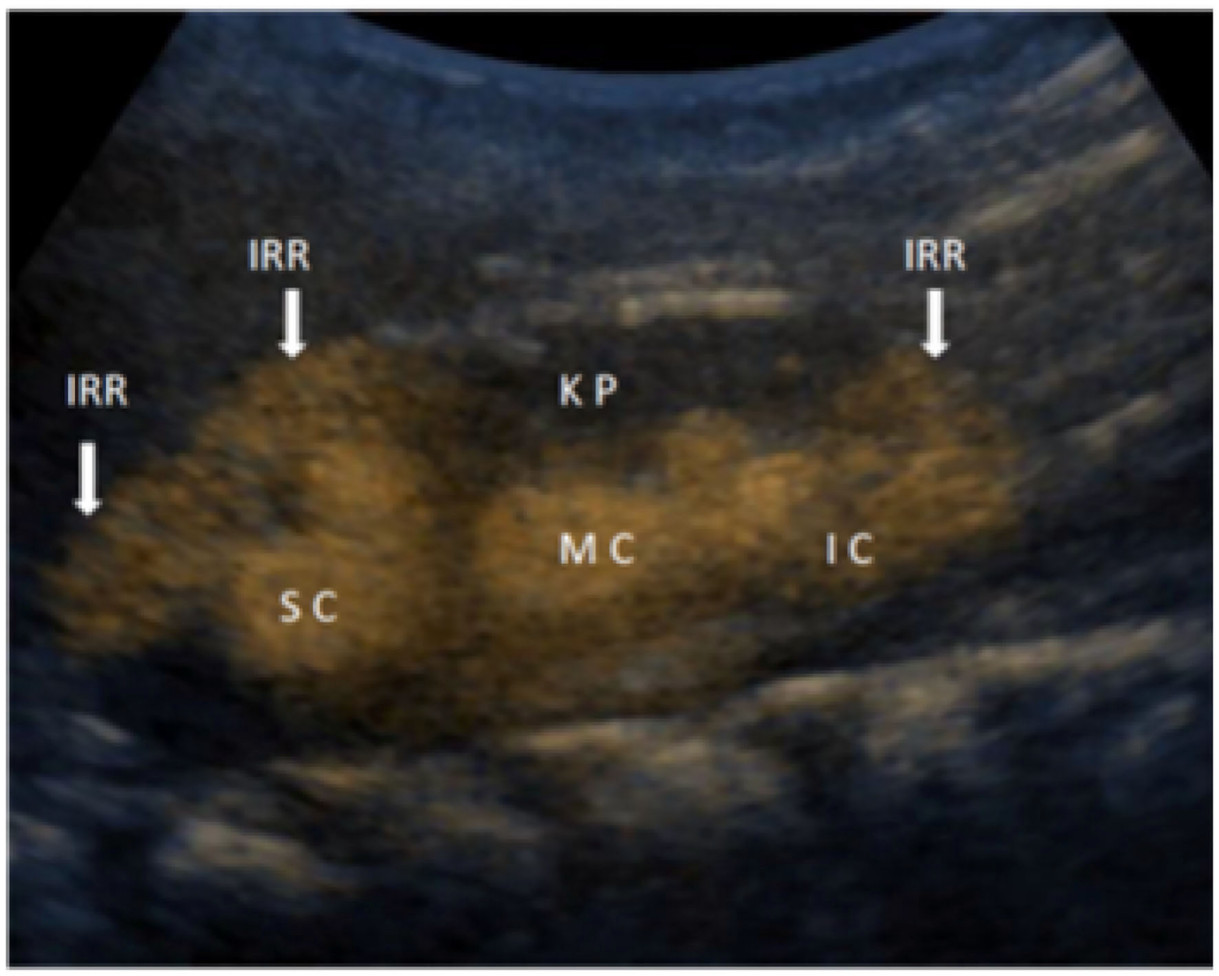
Detailed ceVUS image of the right kidney with intrarenal reflux (IRR) in the area of superior calyces (SC) and in the area of inferior calyces (IC), while in the medial group of calyces (MC), IRR is not present in the kidney parenchyma (KP).

The second generation of UCA solution, consisted of active substance sulfur hexafluoride in the form of microbubbles (Sono Vue, Bracco, Italy) was prepared by instilling 0.5 ml of UCA into 250 ml of saline bag. Then UCA was introduced into the bladder, immediately after preparation *via* a 6F−8F feeding tube or a hydrophilic catheter through the urethra under the pressure of a 70–100-cm column of water until functional bladder capacity was reached or until the child showed the urge to void. The procedure lasts usually 10–20 min, and can be repeated, especially in very young patients. The second generation of UCA is stable and allows the performing of the procedure, without refilling. The additional guaranty for UCA stability is the setting of the US machine to low mechanical index and shift of the US focus out of the observed object. Patients were examined by two experienced pediatric nephrologists on GE Logiq S8 ultrasound machine by probes C 2–9 and 9L, using B-mode and harmonic imaging with mechanical index of 0.06–0.08. The kidneys and bladder were inspected approximately every 10 s and frames were recorded. Some sequences were recorded by video clips. The results were concluded consensually. The IRR appearance is a “smoke-like” extension of UCA, spreading from the renal calyces through the renal parenchyma to the renal periphery (Figure 1).

Seventy patients (41 females and 29 males) with VUR, with expected 140 ureter-renal units (URU) were examined by ceVUS. Out of that number of expected URUs, 37 were excluded from the study due to the following: renal agenesis (1 URU), multicystic dysplasia (2 URUs), duplex channel system (5 URUs), and absence of VUR (29 URUs). Altogether, we analyzed 103 URUs with VURs of grades II–V. Grades I–II were considered non-dilated VURs, grade III as moderately dilated VURs, and grades IV–V as highly dilated VURs. Forty-seven of VURs were right sided, while 56 were left sided. We also recorded the exact locations of IRR in the kidney, distinguishing the occurrence of IRRs in upper, middle, and lower zone, or in combined locations.

### Statistical Analysis

Data are presented as percentages or fractions with 95% confidence intervals when describing a categorical variable or as a median and interquartile range (IQR), as well as range, when presenting a continuous variable. To test trends between ordered categories χ^2^ (Cochrain-Armitage) test for trend was used. To assess differences between continuous variables, the Mann-Whitney-test was used.

*p*-value was interpreted according to ASA Statement on *p*-value from 2016.

## Results

The median age at the diagnosis of VUR was 10 months, (IQR, 4–26 months, range 0–103 months). There were 38 (54.2%) patients with IRR and 32 (45.7%) patients with VUR without IRR. At the time of diagnosis, children with IRR had a median age of 5 months (IQR, 3–14.3), whereas those without IRR had a median age of 15.5 months (IQR, 5–41.5), (*p* = 0.0069). The oldest patient was 7 years and 4 months old when diagnosed with IRR.

Out of all 103 VURs, 55 (53.39%) were of grade II. Eight of them (7,76%) had IRR. Thirty-one (30.09%) were of grade III. Twenty-six of them (25.24%) had IRR, while 17 (16.5%) were of grades IV and V, and all of them (16.5%) had IRR.

Fifty-one of that number (49.51%) had IRR, regardless of the grade of VUR.

Table 1 shows the increase in IRR incidence with VUR severity.

**Table 1:** Incidence of IRR in different VUR stages.

**Grade of VUR**	**Fraction of URUs with IRR**	**% of URUs with IRR**	**95% CI of %**
II	8/55	14.54^a^	7.5–26.1%
III	26/31	83.87^a^	67.3–92.9%
IV and V	17/17	100^a^	81.56–100%
Total	51/103	49.51	40–59%

IRR, intrarenal reflux; URU, ureterorenal unit; VUR, vesicoureteral reflux.

a *χ^2^-test for trend, p < 0.0001*.

**Figure 2:**
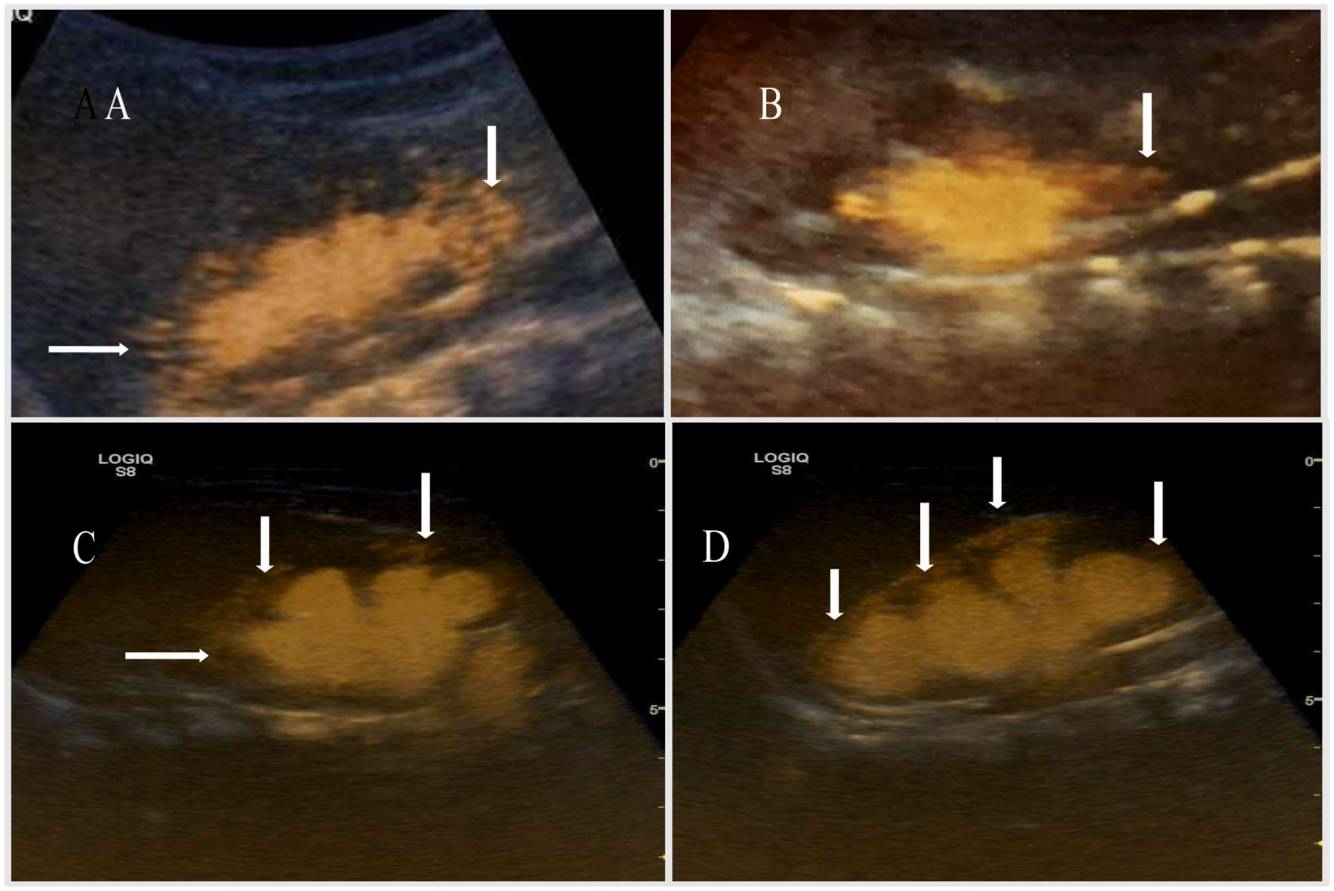
**(A)** The IRR in superior and inferior pole in the patient with VUR grade II (arrows). **(B)** The IRR in the inferior pole in the patient with VUR grade III (arrows). **(C,D)** The IRR into all segments in patient with VURs of grades IV and V (arrows).

The appearance of IRR in different grades of VURs is given in Figures 1, 2.

The significant difference was noticed regarding the occurrence of IRR in renal segments. IRR appeared most frequently in the superior pole, then in the inferior pole, and in the middle segment, while in the all segments together, IRR was noticed less frequently (Table 2).

**Table 2:** Relationship between IRR occurrence and renal segments.

**Renal segment**	**Fraction (%) of IRR**
Superior pole	41/51 (80.39%)^a,b^
Inferior pole	32/51 (62.74%)^a,b^
Middle segment	19/51 (37.25%)^a,b^
All segments	14/51 (27.45%)^b^

a,b *χ2-test for trend, p < 0.0001*.

Since IRR seems to be connected with compound papillae types II and III, which are most abundant in the superior pole, followed by the inferior pole and middle segment of renal parenchyma, we chose to look at relationship between IRR incidence and those segment of renal parenchyma (4, 6). IRR was most common in superior pole parenchyma (80%), followed by inferior pole parenchyma (62.7%), and middle segments (37%), furthermore, simultaneous IRR to all segments of renal parenchyma was the rarest (27%) (*p* < 0.0001) (Table 2).

We also looked at relationship between VUR severity and IRR into specific segments of renal parenchyma (Table 3). The study did not show the significant difference regarding the place of IRR in the superior and inferior poles between the VUR grades, while for IRR position in the middle segment and all segments together did. There was a clear positive trend between VUR grade and frequency of IRR to middle segments and to all of the parenchyma (*p* = 0.0139 and *p* = 0.0104). On the other hand, for superior and inferior pole parenchyma, existence of the trend was more uncertain (*p* = 0.1501, *p* = 0.3145, respectively) (Table 3).

**Table 3:** Relationship between VUR grade and segment of kidney parenchyma affected with IRR.

**Grade of VUR**	**IRR to superior pole, fraction (%)**	**IRR to middle segment, fraction (%)**	**IRR to inferior pole, fraction (%)**	**IRR into all segments, fraction (%)**
II	5/8 (62.5%)^a^	1/8 (12.5%)^b^	4/8 (50%)^c^	0/8 (0%)^d^
III	21/26 (80.76%)^a^	8/26 (30.76%)^b^	16/26 (61.53%)^c^	6/26 (23.07%)^d^
IV and V	15/17 (88.23%)^a^	10/17 (58.8 %)^b^	12/17 (70.58%)^c^	8/17 (47.05%)^d^

*URU, ureterorenal unit; VUR, vesicoureteral reflux*.

a *χ^2^-test for trend, p = 0.1501*.

b *χ^2^-test for trend, p = 0.0193*.

c *χ^2^-test for trend, p = 0.3145*.

d *χ^2^-test for trend, p = 0.0104*.

## Discussion

CeVUS is a sensitive method for the detection of VUR in both sexes. There are some opinions that ceVUS should be applied as a first diagnostic method for VUR only in girls, but not in young boys because of the real possibility of overlooking posterior urethral valve. We share the opinion with some other authors that the posterior urethral valve can be easily diagnosed by ultrasonography as well as by characteristic clinical appearance (17, 24). However, we recommend the use of ceVUS method in young boys, as well. In fact, increased sensitivity is the most probable reason why the VUR of the grade I is rarely diagnosed by ceVUS, as was also shown in our study with no recorded VURs of the grade I. Paradoxically, the VURs of grade I, diagnosed by VCUG have been mostly shown as VURs of grade II and higher on ceVUS, which was reported in a study done by Darge et al., where the 71% of grade I VURs, detected by VCUG had been shown as grade II and higher on ceVUS (17). Similar findings were previously reported by Saraga et al. in patients examined by direct radionuclide cystography (DRNC) and VCUG, where all 17 VURs of grade I detected by VCUG were presented as grade II on DRNC due to higher sensitivity of DRNC (25).

Several studies from the literature, which have been done only by VCUG, reported 1–11% of IRR-associated VURs (5, 7–9, 12, 14), while our study, which has used ceVUS method revealed 49.51% of them. Such high incidence of IRR indicates that UCA of the second generation can easily reach almost all refluxed papillae. The high sensitivity and accuracy of ceVUS regarding the diagnostics of VUR and IRR opens some new questions regarding the role of IRR in kidney function, parenchymal impairment, and potential development of parenchymal scars. Some new studies have to be done to elucidate that new reality.

Thus far, IRR has been referred as a condition which is characteristic for children younger than 4 years, diagnosed mostly after recurrent UTI, and only occasionally diagnosed after that age (5, 9). Our study also showed the similar age distribution, but in our patients, VURs with IRR were diagnosed significantly earlier than VURs without IRR. This might suggest that clinical presentation of patients with IRR could be more earlier and more serious than in patients without IRR. The oldest patient with IRR from our study was 7 years and 4 months old. Possible explanation for that is not clear and needs further elucidation. Maybe, the smaller bladder capacity, and relatively higher frequency of dysfunctional voiding in the younger population can be the cause for increased frequency of IRRs in that population. Namely, incomplete and delayed maturation of the bladder sphincter may be associated with lower urinary tract symptoms (LUTS). In those conditions, recurrent UTIs are common, while high pressure inside the bladder may cause VUR (26). However, the final maturation of kidney tissue starts at the end of prenatal period and continues well into postnatal period. Therefore, incomplete maturity of kidney units and interstitium might explain their higher susceptibility to pathological factors during their first years of life (27, 28).

The entire genesis of the IRR is explained through animal models. According to the results of the study done on piglets, simple papillae and compound papillae type I have not had IRR, while the group of compound papillae types II and III had IRR in 77.66% of cases. The reason for that is specific anatomy of renal papillae and its area cribrosa, as well as inclination of orifices of papillary ducts. While papillary ducts in simple papillae or in type I papillae are opened obliquely through small, slit-like orifices, in compound papillae types II and III orifices are widely opened directly into surface, forming flat, even concave area cribrosa (4). That means that IRR is highly expectable in parts of the kidney with compound papillae types II and III. It was shown that 89.7% of the superior kidney pole consisted of type II or III papillae, likewise 83.34% of the inferior pole consist of the same papillae, while the midzone consisted of 21.7% of the same types of papillae (4). Analogously, distribution of IRR would follow a similar pattern. Similarly, we found that out of all VURs in the superior pole even 80.39% had IRRs, VURs in the midzone had 37.25% of IRR, while VURs from the inferior kidney pole had 62.74% of IRR. That supports the theory that IRR is a consequence of atypical development of renal papillae. Although the IRR was not recorded in the literature in the VUR of grade II, diagnosed by VCUG, our results showed 14.5% of IRRs in grade II VURs, diagnosed by ceVUS. While Schneider et al. noticed IRR in 7.6% of all VUR grade III, and in 30.7% of all VUR grades IV and V on VCUG, our study reported even 83.87% IRRs in grade III, and 100% in grades IV and V on ceVUS (9). All those data underline the need for redefinition of the diagnosis and meaning of IRR in the light of the new diagnostic possibilities, like ceVUS.

Since we know that IRR is based on refluxing papillae, it should not be dependent on VUR grade.

However, it was shown that the number of IRRs rises with the grade of VUR, as it was in our study (5, 9, 13). Therefore, we can also speculate that, besides papillary morphology, the volume and pressure of back-flowed urine surely plays a significant role in the development of IRR. Since VUR is the transporter for possible infectious agents or some other substances excreted from kidneys and concentrated in the urine, it is easy to conclude that renal scar should develop as the end product of interplay of specific papillary shape, volume and pressure of back-flowed urine, and infection. Some studies reported a strong correlation between IRR, diagnosed by VCUG and scar formation, sometimes up to 75% (5–7, 29). Since VCUG discovers only a small portion of IRR, probably only the biggest ones, IRR diagnosed in that way should be associated with parenchymal scars. According to our results, we expect even more IRRs, with a wide spectrum of defects, not only scars, in renal parenchyma due to higher sensitivity of ceVUS compared with VCUG. This will open new possibilities to diagnose, to treat, and to follow-up the process known as VUR.

At the end, we have to note that this study has some limitations including its retrospective nature, relatively small number of patients, and lack of clinical correlation with findings of ceVUS, but we are convinced that we still have representative results, based on sufficient number of data which can improve the diagnosis of VUR.

## Conclusions

In summary, we can say that IRR is one developmental variation, which became highly detectable by ceVUS, which was clearly shown in this study. Based on our study, we can divide VURs in two groups: those with IRR and those without IRR. The distribution of IRRs corresponded to the natural distribution of composed papillae types II and III, while the incidence of IRR increased with severity of VUR. Patients with IRR-associated VUR showed earlier clinical presentation. The results of this study showed that only a minority of non-dilating VURs and majority of dilating VURs would have IRR. We consider IRR as a very significant diagnostic sign. Therefore, further clinical studies may point to the importance of considering IRR in the future classification of VUR

## Data Availability Statement

The raw data supporting the conclusions of this article will be made available by the authors, without undue reservation.

## Ethics Statement

The studies involving human participants were reviewed and approved by Ethical Committee University Hospital of Split. Written informed consent for participation was not required for this study in accordance with the national legislation and the institutional requirements.

## Author Contributions

AS: conceptualization, data curation, formal analysis, investigation, methodology, supervision, validation, visualization, and writing—original draft. AA and MS-B: data curation, formal analysis, investigation, visualization, and writing—original draft. KV: formal analysis, supervision, and writing—original draft. BB: data curation, formal analysis, investigation, and writing—original draft. AP: conceptualization, data curation, investigation, and writing—original draft. MS: conceptualization, data curation, formal analysis, methodology, validation, writing—original draft. All authors contributed to the article and approved the submitted version.

## Conflict of Interest

The authors declare that the research was conducted in the absence of any commercial or financial relationships that could be construed as a potential conflict of interest.

## Abbreviations

ceVUS: contrast-enhanced voiding urosonography
IRR: intrarenal reflux
UCA: ultrasound contrast agent
URU: ureterorenal unit
VUR: vesicoureteral reflux
VCUG: voidingcystourethrography.

## References

[1] Roupakias S Sinopidis X Tsikopoulos G Spyridakis I Karatza A Varvarigou A. Dimercaptosuccinic acid scan challenges in childhood urinary tract infection, vesicoureteral reflux and renal scarring investigation and management. Minerva Urol Nefrol. (2017) 69:144–52. 10.23736/S0393-2249.16.02509-1 27355216

[2] Hewitt I Montini G. Vesicouretral reflux is it important to find? Pediatr Nephrol. (2020). 10.1007/s00467-020-04573-9. [Epub ahead of print]. 32323004

[3] Brodeur AE Goyer RA Melick W. A potential hazard of barium cystography. Radiology. (1965) 85:1080–4. 10.1148/85.6.1080 5892138

[4] Ransley PG Risdon RA. Renal papillary morphology and intrarenal reflux in the young pig. Urol Res. (1975) 3:105–9. 10.1007/BF00256030 1189137

[5] Rolleston GL Maling TM Hodson CJ. Intrarenal reflux and the scarred kidney. Arch Dis Child. (1974) 49:531–9. 10.1136/adc.49.7.531 4852544 PMC1648904

[6] Hannerz L Wikstad I Johansson L Broberger O Aperia A. Distribution of renal scars and intrarenal reflux in children with a past history of urinary tract infection. Acta Radiol. (1987) 28:443–6. 10.1177/028418518702800414 2958060

[7] Uldall P Frøokjaer O Kaas Ibsen K. Intrarenal reflux. Acta Paediatr Scand. (1976) 65:711–5. 10.1111/j.1651-2227.1976.tb18008.x 998230

[8] Cremin BJ. Observations on vesico-ureteric reflux and intrarenal reflux: a review and survey of material. Clin Radiol. (1979) 30:607–21. 10.1016/S0009-9260(79)80003-3 509863

[9] Schneider KO Lindemeyer K Kammer B. Intrarenal reflux, an overlooked entity—retrospective analysis of 1,166 voiding cysturethrographies in children. Pediatr Radiol. (2019) 49:617–25. 10.1007/s00247-018-04330-z 30683961

[10] Rose JS Glassberg KI Waterhouse K. Intrarenal reflux and its relationship to renal scarring. J Urol. (1975) 113:400–3. 10.1016/S0022-5347(17)59492-6 1117509

[11] Fujimatsu A. Diagnosis of intrarenal reflux and its role in pathogenesis of reflux nephropathy in children. Kurume Med J. (2000) 47:109–14. 10.2739/kurumemedj.47.109 10948648

[12] Boubnova J Sergent-Alaoui A Deschênes G Audry G. Evolution and prognosis value of intrarenal reflux. J Pediatr Urol. (2011) 7:638–43. 10.1016/j.jpurol.2010.09.015 20951095

[13] Fukui S Watanabe M Yoshino K. Intrarenal reflux in primary vesicoureteral reflux. Int J Urol. (2013) 20:631–6. 10.1111/iju.12015 23186044

[14] Darge K Trusen A Gordjani N Riedmiller H. Intrarenal reflux: diagnosis with contrast-enhanced harmonic US. Pediatr Radiol. (2003) 33:729–31. 10.1007/s00247-003-1050-2 12928758

[15] Colleran GC Barnewolt CE Chow JS Paltiel HJ. Intrarenal reflux: diagnosis at contrast enhanced voiding urosonography. J Ultrasound Med. (2016) 35:1811–9. 10.7863/ultra.15.09056 27371375

[16] Darge K. Voiding urosonography with ultrasound contrast agents for the diagnosis of vesicoureteric reflux in children. I. Procedure. Pediatr Radiol. (2008) 38:40–53. 10.1007/s00247-007-0529-7 17618429 PMC2292498

[17] Darge K. Voiding urosonography with US contrast agents for the diagnosis of vesicoureteric reflux in children. II. Comparison with radiological examinations. Pediatr Radiol. (2008) 38:54–63. 10.1007/s00247-007-0528-8 17639371

[18] Papadopoulou F Anthopoulou A Siomou E Efremidis S Tsamboulas C Darge K. Harmonic voiding urosonography with a second-generation contrast agent for the diagnosis of vesicoureteral reflux. Pediatr Radiol. (2009) 39:239–44. 10.1007/s00247-008-1080-x 19096835

[19] Duran C Beltrán VP González A Gomez C del Riego J. Contrast-enhanced voiding urosonography for vesicoureteral reflux diagnosis in children. Radiographics. (2017) 37:1854–69. 10.1148/rg.2017170024 29019761

[20] Mane N Sharma A Patil A Gadekar C Andankar M Pathak H. Comparison of contrast-enhanced voiding urosonography with voiding cystourethrography in pediatric vesicoureteral reflux. Turk J Urol. (2018) 44:261–7. 10.5152/tud.2018.76702 29733800 PMC5937646

[21] Ntoulia A Back SJ Shellikeri S Poznick L Morgan T Kerwood J. Contrast-enhanced voiding urosonography (ceVUS) with the intravesical administration of the ultrasound contrast agent Optison™ for vesicoureteral reflux detection in children: a prospective clinical trial. Pediatr Radiol. (2018) 48:216–26. 10.1007/s00247-017-4026-3 29181582

[22] Ključevšek D Battelino N TomaŽič M Kersnik Levart T. A comparison of echo-enhanced voiding urosonography with x-ray voiding cystourethrography in the first year of life. Acta Paediatr. (2012) 101:e235–9. 10.1111/j.1651-2227.2011.02588.x 22211993

[23] Kenda RB Novljan G Kenig A Hojker S Fettich JJ. Echo-enhanced ultrasound voiding cystography in children: a new approach. Pediatr Nephrol. (2000) 14:297–300. 10.1007/s004670050762 10775072

[24] Giordano M Marzolla R Puteo F Scianaro L Carnigella DA Depalo T. Voiding urosonography as first step in the diagnosis of vesicoureteral reflux in children: a clinicalexperience. Pediatr Radiol. (2007) 37:674–7. 10.1007/s00247-007-0499-9 17520246

[25] Saraga M Stanicić A Marković V. The role of direct radionuclide cystography in evaluation of vesicoureteral reflux. Scand J Urol Nephrol. (1996) 30:367–71. 10.3109/00365599609181312 8936625

[26] Tekgul S Stein R Bogaert G Undre S Nijman RJM Quaedackers J. EAU-ESPU guidelines recommendations for daytime lower urinary tract conditions in children. Eur J Pediatr. (2020) 179:1069–77. 10.1007/s00431-020-03681-w 32472266

[27] Hinchliffe SA Sargent PH Howard CV Chan YF van Velzen D. Human intrauterine renal growth expressed in absolute number of glomeruli assessed by dissector method and Cavalieri principle. Lab Invest. (1991) 64:777–84. 2046329

[28] Faa G Gerosa C Fanni D Nemolato S Locci A Cabras T. Marked interindividual variability in renal maturation of preterm infants: lessons from autopsy. J Matern Fetal Neonatol Med. (2010) 23(Suppl):129–33. 10.3109/14767058.2010.510646 20836739

[29] Kim SW Im YJ Hong CH Han SW. The clinical significance of intrarenal reflux in voiding cystourethrography (VCUG). Korean J Urol. (2010) 51:60–3. 10.4111/kju.2010.51.1.60 20414413 PMC2855467

